# Supplementary material for: An improved robust algorithms for fisher discriminant model with high dimensional data
Source: PLoS One. 2025 Jun 12;20(6):e0322741. doi: 10.1371/journal.pone.0322741 (PMC12161537; doi:10.1371/journal.pone.0322741)
Supplement: S1 Table — Calculation of the center of sample class of the partial test set using MCD-Fisher discriminant, OGK-Fisher discriminant and MRCD-Fisher discriminant methods (PDF) [file pone.0322741.s001.pdf]

Supplementary Materials

Table S1: Calculation of partial testing set sample class center by MCD-Fisher discriminant, OGK-Fisher discriminant and MRCD-Fisher discriminant methods.

Variable		Center1			Center2			Center3	
name	MCD	MRCD	OGK	MCD	MRCD	OGK	MCD	MRCD	OGK
UUU	0.02170	0.02400	0.02211	0.03940	0.03180	0.03760	0.04942	0.05658	0.04120
UUC	0.01791	0.01696	0.01819	0.02894	0.03256	0.02922	0.02168	0.02188	0.02171
UUA	0.01625	0.01906	0.01588	0.04731	0.04001	0.04561	0.03861	0.04042	0.03604
UUG	0.01464	0.01589	0.01572	0.00814	0.00524	0.00826	0.02490	0.02637	0.02128
CUU	0.01429	0.01552	0.01523	0.02468	0.02421	0.02357	0.02454	0.02561	0.02323
CUC	0.01201	0.01062	0.01263	0.02328	0.02675	0.02576	0.00526	0.00481	0.00614
CUA	0.00923	0.01041	0.00975	0.04841	0.06052	0.05284	0.01531	0.01587	0.01547
CUG	0.02118	0.01702	0.01989	0.00889	0.00924	0.00969	0.00736	0.00686	0.00747
AUU	0.02471	0.02689	0.02411	0.04892	0.04787	0.04732	0.03945	0.04026	0.03849
AUC	0.02013	0.01790	0.01940	0.03374	0.04064	0.03482	0.01589	0.01534	0.01708
AUA	0.01487	0.01660	0.01371	0.04129	0.04294	0.04067	0.02318	0.02720	0.02311
AUG	0.02489	0.02469	0.02427	0.01383	0.01106	0.01364	0.01938	0.01826	0.02186
GUU	0.01878	0.02085	0.01984	0.01610	0.01248	0.01584	0.01861	0.01657	0.02036
GUC	0.01386	0.01168	0.01347	0.00900	0.01042	0.00888	0.00629	0.00602	0.00608
GUA	0.01284	0.01414	0.01271	0.01995	0.02018	0.02210	0.02018	0.01817	0.02074
GUG	0.01934	0.01751	0.01872	0.00575	0.00478	0.00517	0.00793	0.00644	0.00791
GCU	0.02038	0.02149	0.02140	0.01772	0.01531	0.01823	0.02311	0.02093	0.02515
GCC	0.01839	0.01486	0.01781	0.02446	0.02821	0.02695	0.00672	0.00615	0.00749
GCA	0.02059	0.02133	0.02052	0.02315	0.02551	0.02414	0.01261	0.01033	0.01504
GCG	0.01348	0.00972	0.01232	0.00267	0.00181	0.00229	0.00527	0.00390	0.00595
CCU	0.01261	0.01374	0.01409	0.01412	0.01235	0.01373	0.01411	0.01297	0.01689
CCC	0.00858	0.00797	0.00987	0.01521	0.01711	0.01662	0.00643	0.00614	0.00710
CCA	0.01416	0.01549	0.01470	0.01980	0.02442	0.02177	0.01098	0.01102	0.01152
CCG	0.00956	0.00701	0.00903	0.00208	0.00167	0.00195	0.00479	0.00382	0.00559
UGG	0.01436	0.01443	0.01534	0.00451	0.00321	0.00459	0.01533	0.01689	0.01609
GGU	0.01873	0.01919	0.01937	0.01248	0.00915	0.01317	0.01912	0.01498	0.02239
GGC	0.01744	0.01437	0.01655	0.01293	0.01424	0.01232	0.00474	0.00460	0.00483
GGA	0.01691	0.01862	0.01800	0.02328	0.02414	0.02354	0.01995	0.02002	0.02455
GGG	0.01088	0.01071	0.01184	0.00893	0.00809	0.00883	0.00931	0.00852	0.01153
UCU	0.01360	0.01518	0.01450	0.01674	0.01383	0.01619	0.02978	0.03279	0.02495
UCC	0.00990	0.00950	0.01085	0.01483	0.01662	0.01462	0.01206	0.01235	0.01212
UCA	0.01510	0.01650	0.01498	0.02390	0.02669	0.02371	0.01906	0.01835	0.01732
UCG	0.00700	0.00586	0.00711	0.00270	0.00177	0.00210	0.00717	0.00696	0.00720
AGU	0.01189	0.01297	0.01189	0.00718	0.00417	0.00756	0.01457	0.01611	0.01442
AGC	0.01213	0.01144	0.01174	0.00758	0.00874	0.00885	0.00296	0.00303	0.00351
ACU	0.01606	0.01767	0.01671	0.01781	0.01682	0.01785	0.02059	0.02051	0.02194

ACC	0.01579	0.01349	0.01575	0.02302	0.02666	0.02358	0.00766	0.00657	0.00901
ACA	0.01847	0.02026	0.01829	0.03145	0.03712	0.03320	0.00983	0.00889	0.01248
ACG	0.00979	0.00837	0.00949	0.00289	0.00242	0.00236	0.00433	0.00381	0.00502
UAU	0.01981	0.02168	0.01953	0.01959	0.01541	0.01898	0.04167	0.04739	0.03423
UAC	0.01537	0.01463	0.01511	0.01417	0.01613	0.01442	0.00957	0.00828	0.00878
CAA	0.02006	0.02203	0.02053	0.02006	0.02048	0.02000	0.02509	0.02449	0.02529
CAG	0.02049	0.01846	0.02019	0.00370	0.00254	0.00283	0.00831	0.00827	0.00789
AAU	0.02579	0.02879	0.02497	0.02100	0.01691	0.02098	0.03697	0.04009	0.03462
AAC	0.02212	0.02128	0.02164	0.02075	0.02430	0.02135	0.01085	0.01085	0.01153
UGU	0.00798	0.00877	0.00883	0.00457	0.00308	0.00353	0.00912	0.00932	0.00987
UGC	0.00763	0.00754	0.00811	0.00392	0.00438	0.00364	0.00253	0.00215	0.00318
CAU	0.01144	0.01225	0.01170	0.00975	0.00820	0.00875	0.02448	0.02530	0.02177
CAC	0.01015	0.00948	0.01029	0.01345	0.01601	0.01364	0.00604	0.00595	0.00618
AAA	0.03485	0.03787	0.03337	0.02625	0.02294	0.02447	0.04598	0.04792	0.04177
AAG	0.02712	0.02639	0.02680	0.00440	0.00252	0.00381	0.01309	0.01321	0.01328
CGU	0.00938	0.00876	0.00957	0.00339	0.00261	0.00370	0.01346	0.01173	0.01468
CGC	0.01076	0.00758	0.00949	0.00285	0.00292	0.00263	0.00393	0.00360	0.00406
CGA	0.00580	0.00580	0.00617	0.00908	0.01034	0.00921	0.01378	0.01295	0.01444
CGG	0.00551	0.00456	0.00559	0.00153	0.00125	0.00111	0.00520	0.00548	0.00419
AGA	0.01367	0.01540	0.01339	0.00685	0.00336	0.00602	0.01603	0.01649	0.01570
AGG	0.00875	0.00880	0.00900	0.00129	0.00024	0.00088	0.00467	0.00518	0.00482
GAU	0.03127	0.03328	0.03039	0.00919	0.00612	0.00954	0.03073	0.02932	0.03301
GAC	0.02439	0.02173	0.02363	0.00972	0.01102	0.00890	0.00821	0.00810	0.00785
GAA	0.03499	0.03661	0.03353	0.01771	0.01653	0.01863	0.03803	0.03480	0.03969
GAG	0.02668	0.02503	0.02603	0.00478	0.00357	0.00418	0.01137	0.01068	0.01289
UAA	0.00164	0.00168	0.00163	0.00237	0.00216	0.00251	0.00105	0.00087	0.00130
UAG	0.00060	0.00057	0.00064	0.00057	0.00062	0.00050	0.00023	0.00016	0.00070
UGA	0.00134	0.00111	0.00122	0.02197	0.02561	0.02209	0.00113	0.00110	0.00098
